# Mitochondrial checkpoint for interferon responses in macrophages

**DOI:** 10.37349/etat.2026.1002399

**Published:** 2026-08-19

**Authors:** Andreas O. Mieland, Oliver H. Krämer

**Affiliations:** Fondazione Policlinico Universitario Agostino Gemelli IRCCS, Italy; Institute of Toxicology, University Medical Center of Johannes Gutenberg University, 55131 Mainz, Germany

**Keywords:** acetylation, efferocytosis, histone deacetylase (HDAC), JAK-STAT, gene expression, interferon, ISG15, macrophage

## Abstract

A functional immune system is a key antagonist of cancer cell growth. Cytokines such as interferons (IFNs) promote the onset of inflammation, turn cells into an anti-viral state, and shape the dynamic tumor-immune cell interactome. Recent work illustrates how type I IFNs contribute to the resolution of inflammatory conditions. This involves macrophage-mediated efferocytosis for the clearance of apoptotic cells and the intrinsic capacity of type I IFNs to restrict their own autocrine signaling loops via the IFN-stimulated gene 15 (ISG15) protein. We discuss how this may affect tumor cells and how acetylation-dependent processes can affect the phosphorylation-dependent signaling cascades that augment IFN-dependent gene expression.

## Macrophage metabolism beyond the Warburg effect

A central concept of immunometabolism is that macrophage activation, specifically through extracellular toll-like receptor (TLR) stimulation, triggers a Warburg-like metabolic shift. In this model, cells prioritize aerobic glycolysis to gain ATP energy equivalents and suppress mitochondrial respiration (aerobic oxidative phosphorylation) despite it being the pathway that yields more ATP. This metabolic reprogramming can provide energy equivalents and biological precursors for pro-inflammatory effector functions more rapidly [1]. Cancer cells often hijack this metabolic adaptation to achieve rapid cell proliferation. The tumor microenvironment (TME) following this switch is commonly nutrient-deprived, promoting inflammation and secondary necrosis [2].

Here we focus on the recent article by Dunphy et al. [3], who found that intracellular, cytosolic nucleic acid sensor proteins, such as stimulator of interferon genes (STING) and mitochondrial antiviral-signaling (MAVS) protein, induce different metabolic adaptations in primary bone marrow-derived murine macrophages. Unlike TLR agonists, these sensors activate a signaling axis that preserves mitochondrial respiration while specifically modulating the mitochondrial membrane potential (MMP) [3]. This distinction is critical as it suggests that the innate immune system possesses a “metabolic toolkit” that is adapted to the specific nature of the threat, moving the field toward a deeper understanding of how specific stimuli influence cellular fate.

## Mechanistic core: interferon (IFN)-mitochondria axis

The type I IFNs, IFNα and IFNβ, and the plasma membrane-bound IFN-α/β receptor (IFNAR) are primary drivers of the specific metabolic state that is shown by Dunphy et al. [3]. These cytokines were first identified as auto- and paracrine mediators of antiviral defense and are highly appreciated regulators of immune responses in health and disease [4, 5]. IFNα/IFNβ promote IFNAR signaling to induce or augment the expression of IFN-stimulated genes (*ISGs*). These include the *ISG15* gene and its product ISG15. This abundant, ubiquitin-like protein has an impact on various steps of tumorigenesis, cancer therapy, and inflammatory processes [6–8]. ISG15 can be attached covalently to lysine residues in several target proteins [6]. Proteomics identified mitochondrial targets of ISG15, including subunits of complex V, which is responsible for ATP synthesis in mitochondria. Conjugation with ISG15, also termed ISGylation, increases complex V activity, leading to enhanced ATP production and a simultaneous lowering of MMP values [3].

Not only protein-bound but also free ISG15 can exert biological functions [6]. Gain-of-function assays in which conjugation-competent (ISG15-GG) and incompetent (ISG15-AA) mutants were exogenously expressed in ISG15 null macrophages revealed that the covalent attachment of ISG15 was essential for IFN-induced mitochondrial adaptations [3]. Thus, this posttranslational modification is a molecular mediator of the observed phenotypes. These data agree with recent findings that position ISGylation as a central mediator of metabolic modulation upon STING activation. Type I IFNs promote fatty acid oxidation, and ISG15 dampens mitochondrial fatty acid oxidation in a negative feedback loop. This attenuates inflammation to maintain balanced immune responses [9].

## Mechanistic impact of ISG15 on efferocytosis and viral replication

**Figure 1:**
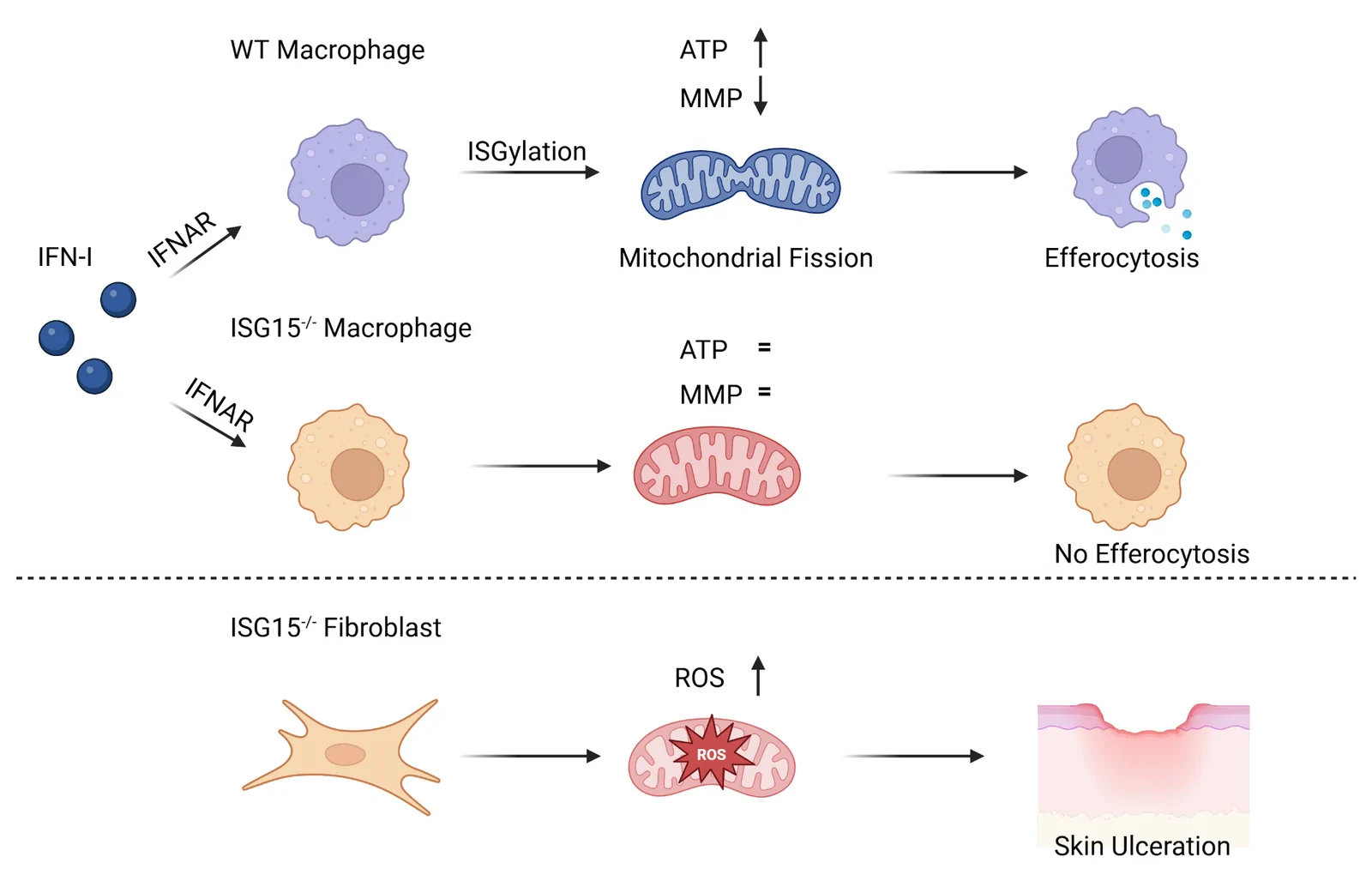
**Schematic overview of cell-type-specific and context-dependent metabolic programming by ISG15.** Wild-type (WT) macrophages respond to IFNAR stimulation with elevated ISGylation, which drives a metabolic shift toward increased ATP production and mitochondrial fission. This response simultaneously lowers the MMP, ultimately facilitating enhanced efferocytosis and resulting in improved immune resolution and decreased secondary necrosis. In ISG15-deficient macrophages, these homeostatic metabolic adaptations are absent, leading to impaired clearance of apoptotic debris followed by an inactive tumor microenvironment (TME) and increased secondary necrosis (upper). ISG15-deficient human fibroblasts exhibit chronically elevated ROS levels and dysregulated extracellular matrix homeostasis. This tissue-specific failure results in the characteristic skin ulcerations observed in human ISG15 deficiency, underscoring the divergent roles of the ISG15-mitochondrial axis in immune resilience versus structural integrity (lower). IFN: interferon; IFNAR: interferon-α/β receptor; ISG: interferon-stimulated gene; MMP: mitochondrial membrane potential; ROS: reactive oxygen species; TME: tumor microenvironment. Created in BioRender. Mieland, A. O. (2026) https://biorender.com/zjg3x4f.

The above-mentioned metabolic shifts are linked to efferocytosis, the clearance of apoptotic cells by immune cells. The IFN-I-induced decrease in MMP directly enhances the efferocytic capacity of macrophages. This suggests that the IFN-I response is not just a pro-inflammatory antiviral signal, but also a preparatory signal for a resolution of inflammatory responses. The physiological relevance of this mechanism was validated in the thymus, a site of high basal cell turnover by programmed cell death (apoptosis). In ISG15-deficient mice, thymic macrophages were unable to clear dying cells, leading to an accumulation of apoptotic debris. This finding positions ISG15 as a key homeostatic regulator, ensuring efferocytosis to prevent secondary necrosis and chronic inflammation [3] (Figure 1).

The double-stranded DNA *vaccinia* virus is a member of the Poxviridae family. It was used as the smallpox vaccine and is related to the *monkeypox* virus, which gave rise to a recent outbreak of concern [10]. *Vaccinia* virus is used to study IFN-dependent antiviral mechanisms. Auricular infections of wild-type mice and mice lacking ISG15 or the mitochondrial zinc metallopeptidase OMA1 surprisingly showed that *Isg15*^–/–^ and *Oma1*^–/–^ mice had lower vaccinia virus titers than wild-type mice. Infected *Isg15*^–/–^ ears displayed a slight increase in monocytes and neutrophils as well as a higher frequency of late-apoptotic neutrophils and monocytes, suggesting a potential defect in apoptotic cell clearance. Bone marrow chimera experiments further showed that mice reconstituted with *Isg15*^–/–^ or *Oma1*^–/–^ hematopoietic cells exhibited reduced vaccinia virus titers at day 7 [3]. Hence, such enhanced viral control appears mediated by the hematopoietic compartment and stems from the increased IFN-dependent target gene induction in *Isg15*^–/–^ and *Oma1*^–/–^ mice. Nonetheless, ISG15 and OMA1 differentially affect alterations in the MMP of type I IFN-treated macrophages, with only *ISG*15^–/–^ cells differing from wild-type cells [3]. Congruent with this, a link between elevated *ISG15* expression and the resolution of induced immunological responses in murine *lymphocytic choriomeningitis* virus-infected mice was found. Their macrophages had increased fatty acid oxidation that tied in with an enhanced expression of IFNβ and its target proteins signal transducers and activators of transcription-1/-2 (STAT1/STAT2). A subsequent time-delayed accumulation of ISG15 was linked to attenuated fatty acid oxidation and less expression of pro-inflammatory factors [9].

**Figure 2:**
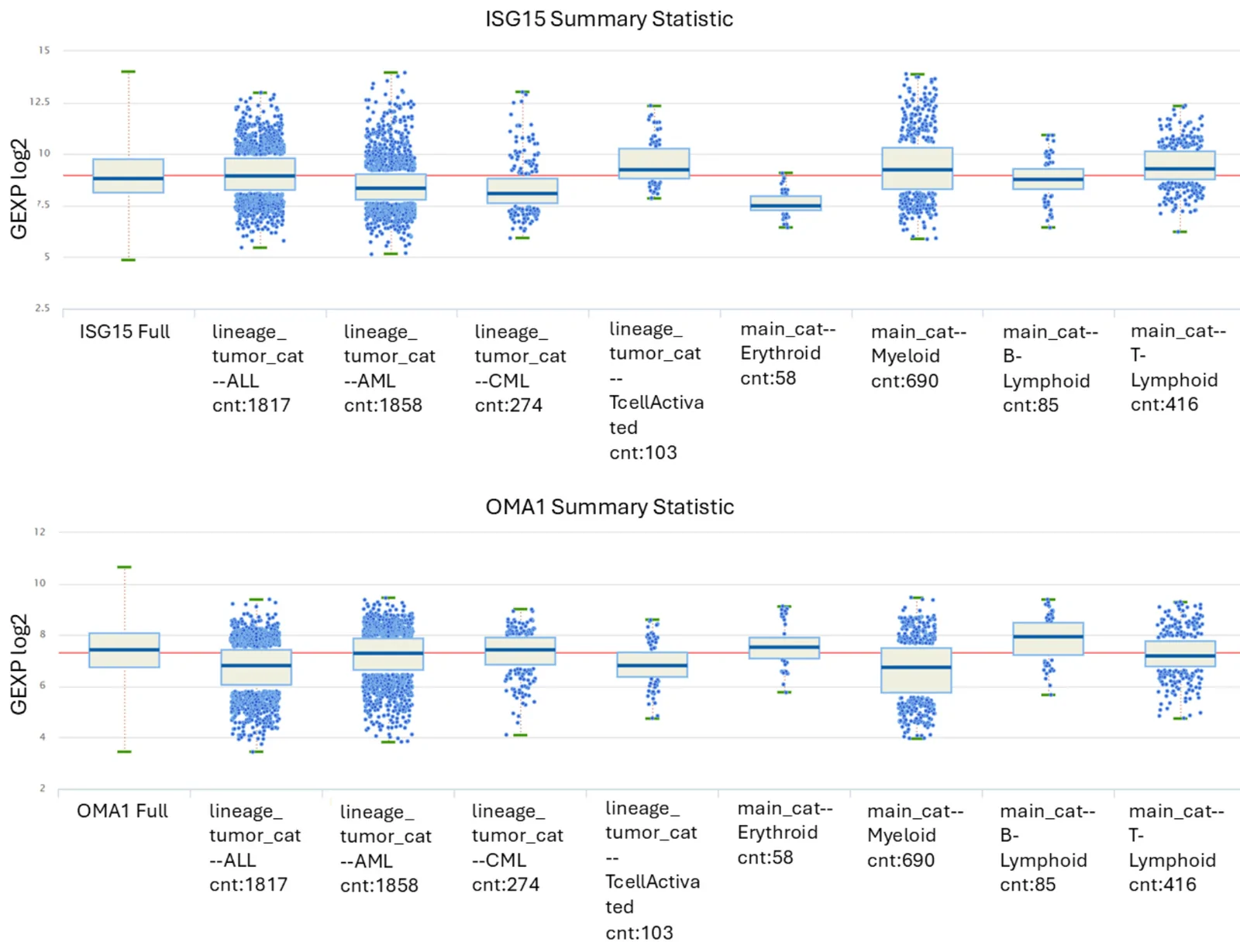
**mRNA expression levels of *ISG15* and *OMA1* in normal and transformed blood cells.** The HEMAP database (HEMAP: Online Resource for Interactive Exploration and e-Staining of Hematopoietic Cancer Data; Blood Cancer/AML Maps and Exploration) was used to assess the mRNA expression levels of *ISG15* and *OMA1* in the indicated cell types. AML: acute myeloid leukemia; CML: chronic myeloid leukemia; cnt: counted number of independent samples; GEXP log2: log_2_-transformed gene expression.

How deficiencies in the *ISG15* and *OMA1* genes affect T cells, especially cytotoxic T lymphocytes that induce apoptosis of virus-infected cells, and B cells should be evaluated in future studies. This might be particularly relevant for DNA viruses that possess the capacity to integrate into the host genome to drive viral carcinogenesis [11]. Understanding the ISG15-OMA1-mitochondria axis in this context could reveal how metabolic checkpoints influence the elimination of pre-cancerous cells. Furthermore, exploring the levels of proteins involved in these processes in both normal and transformed cell types may provide new biomarkers or therapeutic targets for treating neoplastic malignancies. For example, a comparison of the expression levels of *ISG15* and *OMA1* in human leukemic cells using the HEMAP database shows that these are differentially expressed among normal and leukemic hematopoietic cell types (Figure 2). Whereas *ISG15* levels are lower in acute myeloid leukemia (AML) cells and chronic myeloid leukemia (CML) cells than in normal myeloid cells, *OMA1* levels show an opposite trend (Figure 2).

To formally prove the findings and derived hypotheses for ISG15 and OMA1, cross-breeding with mice lacking upstream IFN signaling components would be necessary. Upon IFNAR binding, IFNα and IFNβ induce Janus kinases (JAKs) that phosphorylate STATs. Phosphorylated STAT1, STAT2, and the associated IFN regulatory factor-9 build the IFN-stimulated gene factor-3, which is the essential transcription factor for genes that program cells to an antiviral state [12, 13]. Whether other IFNs such as the type II IFN IFNγ and the type III IFNs have an impact on the IFN-ISG15/OMA1 interplay requires additional experimentation.

Work preceding the analyses by Dunphy et al. [3] and Gupta et al. [9] showed that replication of *vaccinia* virus in bone marrow-derived macrophages induced reactive oxygen species (ROS) [14]. These mitochondria-derived byproducts of cellular metabolism are signaling intermediates that contribute to beneficial and detrimental immune responses [15]. Following *vaccinia* virus infection, *Isg15*^+/+^ bone marrow-derived macrophages exhibited significantly greater increases in mitochondrial respiration, ATP production, and ROS levels than *Isg15*^–/–^ macrophages. This was associated with increased nitric oxide production and arginase-1 activation, concomitant with a decrease in viral titers [14]. Moreover, even a *vaccinia* virus, which is unable to grow in wild-type cells, caused fatal lung inflammation in ISG15 null mice [16]. On the other hand, ISG15 supports the dissemination of infection-competent *vaccinia* virus particles [17]. The impact of ISG15 on how cells control virus infections might involve additional levels of complexity. This may be complicated by the induction of apoptosis and other cell death modes as well as differences in mouse and virus strains.

The translation to primary human disease contexts remains an area for further investigation. It will be particularly interesting to see if and how ISG15 and its interplay with IFN-associated metabolic changes affects more severe, systemic viral infections and how this might become therapeutically relevant. Species-specific differences must be considered in such analyses. For example, free and conjugated ISG15 can act antiviral and *influenza B* virus blocks this specifically in human and non-human primate cells [18].

## Negative feedback loop via *OMA1*

Mice lacking the *Oma1* or *Isg15* genes display increased IFN-stimulated histone acetylation and enhanced mRNA and protein expression of the ISGs *Ifit3* and *Ly6A/E*. A drop in MMP triggers the mitochondrial protease OMA1, which induces mitochondrial fission. This structural change reduces physical contacts between the mitochondria and the endoplasmic reticulum (ER). Because these contact sites are necessary for sustained IFN signaling, mitochondrial fission could function as a metabolic repressor that limits further induction of *ISGs* [3] (Figure 1). Congruently, the ISG15 protein dampens fatty acid oxidation and type 1 IFN responses in bone marrow-derived macrophages [9]. Though this resulted in enhanced viral control in acute settings, the loss of the metabolic brake suggests a potential risk for the development of chronic autoinflammatory conditions—if the response cannot be properly terminated. This can be deduced from human systemic lupus erythematosus patients. Monocytes from patients with this antibody-driven interferonopathy exhibit increased ISG15 expression and reduced fatty acid oxidation compared with monocytes from healthy donors [9].

## Acetylation as putative regulator of *ISG15*-dependent mechanisms?

Dunphy et al. [3] considered that sirtuins, NAD^+^-dependent enzymes that control the acetylation of histones and non-histone proteins, mediate the increased ISG induction in *Isg15*- and *Oma1*-deficient murine bone marrow-derived macrophages that were stimulated with IFN-β. A non-significant change in the NAD^+^/NADH ratio was interpreted as sirtuins not being important for this process. However, such measurement does not consider the expression levels and posttranslational modification-dependent activities of sirtuins. Both may be involved in the IFN-modulated efferocytosis phenotype. By deacetylating STAT1 at K673 and STAT3 at K679/K685/K707/K709, murine SIRT1 limits the activating phosphorylation of STAT1 at Y701 and STAT3 at Y705. Accordingly, decreased levels of SIRT1 in primary *Sirt1*^+/–^ peritoneal macrophages and *Sirt1*^+/–^ mice promote IFN-induced signaling and antiviral defense [19]. In addition, the acetylation of STAT1 by the acetyltransferase CREB-binding protein (CBP) and its deacetylation by the class I histone deacetylase (HDAC) HDAC3 and other HDACs in murine and human cells [13, 20] are putative regulators of IFN-regulated MMP and efferocytosis. This might additionally involve an intricate interplay between class I HDACs and the JAK-STAT signaling cascade [13, 21]. Given that class I HDACs critically drive both oncogenic pathways and immune evasion, targeting these epigenetic modifiers with specific HDAC inhibitors may represent a feasible therapeutic strategy to reprogram the TME and suppress cancer progression [22, 23].

## Broader impact and future perspectives

Why is a deeper understanding of the above mechanism relevant? The term efferocytosis comes from Latin, with *effero* meaning to carry to the grave. This specialized phagocytic clearance of apoptotic cells maintains tissue health as it prevents inflammation. If efferocytosis fails, dead cells accumulate, and secondary necrosis can occur, releasing inflammatory signals. Such unbalanced cell fate might contribute to autoimmune diseases such as myasthenia gravis and systemic lupus erythematosus [9, 24]. In CML, tumor-secreted factors like lactotransferrin suppress efferocytosis by reducing CD36 expression on macrophages, thereby facilitating immune evasion [25]. However, efferocytosis in the leukemia microenvironment can be a double-edged sword. Although it prevents necrosis, continuous debris clearance often triggers tolerogenic M2-polarization of macrophages, which increases immune checkpoints that suppress T cells [26]. In line with this, clinical scoring models in AML identified distinct efferocytosis-related subtypes, in which a high efferocytosis score strongly correlates with immune exhaustion and poor patient prognosis [27]. In addition, ISGylation can promote excessive inflammation. This was observed for intestinal inflammation that led to IFN-induced ROS and cytokine expression, resulting in colitis-associated colon cancer in mice [28]. Targeting the feedback of ISG15 and OMA1 on IFN-dependent gene expression may not be easily feasible because of the central roles that IFNs play. Besides, it should be considered that the therapeutic potential of targeting the ISG15-mitochondrial axis is significantly constrained by the functional variety of this molecule across different tissue compartments. Clinical evidence from human patients with *ISG15* gene deficiency reveals that its absence leads to severe ulcerating skin lesions and a collapse of connective tissue integrity. This pathology occurs because ISG15 is important for the structural homeostasis of dermal fibroblasts and keratinocytes, where it maintains the expression of critical adhesion molecules like desmogleins and prevents the overactivation of matrix metalloproteinases. Furthermore, there are elevated ROS levels and reduced expression of ROS scavengers in *ISG15*-deficient fibroblasts. As a systemic attempt to modulate ISG15 might trigger oxidative stress and impair cell migration, underscoring the necessity of a highly cell-type-specific approach to mitigate these risks [29] (Figure 1).

In this context, a key limitation of the study by Dunphy et al. [3] is that mechanistic insights on the ISG15-OMA1-mitochondria axis were collected in murine bone marrow-derived macrophages. Although database analyses like HEMAP offer translational hints, future studies should address whether human macrophage subsets in the complex TME recapitulate the metabolic checkpoints observed in mouse models. Depending on the outcome of such studies, translation into therapies considering immunometabolism might be realized.

The hope in IFNs as clinically useful pro-inflammatory, immune-stimulating molecules against tumor cells has not been fulfilled. Deeper knowledge of how IFNs are controlled in vivo and how this is connected to metabolic adaptation may allow an improved usage of IFNs. Further insights into the molecular mechanisms that control efferocytosis as a function of altered MMP may deliver small molecules that boost anti-tumor effects of IFNs. Nevertheless, therapeutic interventions must be carefully balanced. Excessive JAK-STAT signaling is known to contribute to tumorigenesis in organs like the colon and acts as a driver of myeloproliferative neoplasms, such as those caused by the *JAK2V617F* mutation [13, 21]. Targeting the ISG15-mitochondria axis may be a safer approach to modulate immune responses that circumvents overactive IFN signaling.

In addition, deeper insights into how ISG15 regulates cellular and organismal processes could be derived from the analysis of the E2 ubiquitin conjugating enzyme UBCH8. This enzyme is induced upon HDAC inhibitor-induced chromatin hyperacetylation, and the *UBE2L6* gene, which encodes UBCH8, harbors IFNα/IFNβ-activated *ISRE* and IFNγ-activated *GAS* sequences [13, 21]. This and the finding that UBCH8 can conjugate not only ubiquitin but also ISG15 [30] link UBCH8 to the acetylation- and cytokine-induced control of ISG15.

Stimulus-specific metabolism establishes that metabolic reprogramming is not a unidirectional process, encouraging future research to look beyond the Warburg effect in immune contexts (Table 1). It highlights the role of mitochondrial morphology and organelle-ER communication as active regulators of nuclear gene expression rather than passive metabolic consequences. Furthermore, the IFNAR-ISG15-OMA1 axis offers new potential therapeutic targets for the still incompletely exploited applications of IFNs [4, 5]. In autoinflammatory diseases, pharmacological modulation of this axis could promote efferocytosis and resolve persistent inflammation. In oncology, transiently inhibiting the OMA1-mediated metabolic brake could potentially boost the potency of the innate immune response. By identifying the mitochondria as a dynamic rheostat for the immune response, Dunphy et al. [3] provided a foundational framework in the study of innate immunity and inflammation resolution.

**Table 1:** Comparison between toll-like receptor (TLR) signaling and new data summarized herein.

**Feature**	**Classic TLR signaling**	**Cytosolic nucleic acid sensing (Dunphy et al. [3])**
Metabolism	Glycolytic switch (Warburg effect)	Intact OXPHOS (mitochondrial respiration)
ATP production	Low	High
Mitochondria	Suppressed/Dysfunctional	Active, but with low membrane potential (MMP)
Key regulator	HIF-1α/Lactate	ISG15/ISGylation
Morphology	Variable	OMA1-mediated fission
Outcome	Pro-inflammatory persistence	Efferocytosis & resolution

## Conclusions

The classical paradigm of the Warburg effect as a unidirectional metabolic switch during macrophage activation is expanding. The integration of the type I IFN-ISG15-OMA1 axis demonstrates that mitochondria are dynamic metabolic hubs, utilizing organelle morphology and post-translational modifications to actively tune nuclear gene expression and control complex immunological outcomes. While this mitochondrial checkpoint is vital to maintain cellular homeostasis and resolve temporary inflammatory stress, its chronic dysregulation can shift the balance from protective immunity to severe pathological states. Ultimately, deciphering the precise molecular wiring of these organelle-driven feedback loops will be instrumental in reshaping our understanding of immunometabolism. Such insights may unlock new dimensions for targeted therapeutic interventions.

## Abbreviations

AML: acute myeloid leukemia
CML: chronic myeloid leukemia
ER: endoplasmic reticulum
HDAC: histone deacetylase
IFN: interferon
IFNAR: interferon-α/β receptor
ISG: interferon-stimulated gene
JAKs: Janus kinases
MMP: mitochondrial membrane potential
ROS: reactive oxygen species
*STING*: stimulator of interferon genes
TLR: toll-like receptor
TME: tumor microenvironment

## Declarations

### Availability of data and materials

All data supporting the findings of this review are included within the article and its references. Additional data on HEMAP can be found in [31].

### Copyright

© The Author(s) 2026.
